# On the Effect of Sodium Chloride and Sodium Sulfate on Cold Denaturation

**DOI:** 10.1371/journal.pone.0133550

**Published:** 2015-07-21

**Authors:** Andrea Pica, Giuseppe Graziano

**Affiliations:** 1 Dipartimento di Scienze Chimiche, Università degli Studi di Napoli Federico II, Complesso Universitario di Monte Sant’Angelo, Via Cintia – 80126 Napoli, Italy; 2 Istituto di Biostrutture e Bioimmagini, CNR, Napoli, Italy; 3 Dipartimento di Scienze e Tecnologie, Università del Sannio, Via Port’Arsa 11–82100 Benevento, Italy; Università di Napoli Federico II, ITALY

## Abstract

Both sodium chloride and sodium sulfate are able to stabilize yeast frataxin, causing an overall increase of its thermodynamic stability curve, with a decrease in the cold denaturation temperature and an increase in the hot denaturation one. The influence of low concentrations of these two salts on yeast frataxin stability can be assessed by the application of a theoretical model based on scaled particle theory. First developed to figure out the mechanism underlying cold denaturation in water, this model is able to predict the stabilization of globular proteins provided by these two salts. The densities of the salt solutions and their temperature dependence play a fundamental role.

## Introduction

It is widely recognized that globular proteins undergo cold denaturation in aqueous media [1], as further confirmed in the last years by means of detailed experimental studies [2, 3]. Careful analysis of NMR and CD investigations [4, 5] has shown that: (1) yeast frataxin, Yfh1, undergoes cold denaturation at a temperature above 0°C, (2) the transition is exothermic and reversible, (3) the two denatured states (obtained upon cold and hot denaturation, respectively) are very similar from a structural point of view [6].

Yfh1 is a metal-binding protein and its conformational stability is strongly dependent on the presence of salts [7, 8]. In particular, Yfh1 binds divalent cations and even very low concentrations of the latter have very large effects on its stability. However, it has been shown that also low concentrations of salts of monovalent cations, not binding the protein, such as NaCl and Na_2_SO_4_, significantly increase the conformational stability of Yfh1 [8]. Specifically: (a) the hot denaturation temperature, Td,hot, passes from 30°C in water (10 mM HEPES buffer, pH 7.5), to 40°C in 100 mM NaCl, and 48°C in 100 mM Na_2_SO_4_; (b) the cold denaturation temperature, Td,cold, passes from 7°C in water (10 mM HEPES buffer, pH 7.5) to values significantly lower than -20°C in both 100 mM NaCl and 100 mM Na_2_SO_4_; (c) there is also a marked increase in the values of the denaturation Gibbs energy at the temperature of maximal stability, ΔGd(Tmax) [8]. These findings suggest that both salts affect the conformational stability of Yfh1 not only by means of ionic strength effects, but also as a consequence of a change in a basic property of the solvent water.

An approach grounded in statistical thermodynamics has provided a consistent mechanism for the dependence upon temperature of the conformational stability of globular proteins in water [9, 10]. The reliability of this approach to explain the occurrence of cold denaturation has been supported by direct MD simulations in detailed water models [11]. Two solvent properties play a fundamental role: the water density with its peculiar temperature dependence at 1 atm, and the small diameter of water molecules. This theoretical approach is able to provide, with no *ad hoc* assumptions, a rationalization of the experimental findings on Yfh1, by simply taking into account the density increase caused by the addition to water of small amounts of NaCl and Na_2_SO_4_.

## Theoretical Approach

Two macro-states are accessible to protein molecules: the ensemble of native conformations, N-state, and the ensemble of denatured conformations, D-state. According to the theoretical approach [9, 10, 12], the denaturation Gibbs energy change (ΔGd) in both water and aqueous salt solutions is given by:

$$\Delta G_{d}=[\Delta G_{c}\left(D\right)-\Delta G_{c}\left(N\right)]-T\cdot \Delta S_{conf}+[E_{a}\left(D\right)-E_{a}\left(N\right)+\Delta E_{a}\left(intra\right)]$$ (1)

where ΔGc(D) and ΔGc(N) are the Gibbs energy changes associated with the creation in aqueous media of the cavity hosting the D-state and N-state, respectively; ΔSconf represents the increase in conformational entropy of the protein chain upon denaturation; Ea(D) and Ea(N) are the energies obtained by taking into account all the interactions waters and ions establish with the protein in the D-state and N-state, respectively; ΔEa(intra) is the intra-protein energy loss upon denaturation. It is worth noting that in Eq (1) no contribution from the structural rearrangement of water H-bonds has been considered. For the latter process an almost complete enthalpy-entropy compensation holds [13, 14]. Furthermore, it can be assumed that the second square bracket in Eq (1), labelled ΔE, is close to zero. This assumption relies on the consideration that the sum of the intra-molecular interactions in the N-state and the inter-molecular interactions of N-state with waters are almost entirely counterbalanced by the inter-molecular interactions of D-state with waters (for a more detailed discussion, see ref. [10] and S1 Text). This assumption is considered to hold also in the case of aqueous solutions of NaCl and Na_2_SO_4_. It is firmly established that the Na^+^, Cl- and SO42- ions preferentially interact with waters [15, 16], and so should be excluded from the protein solvation shell of both the N-state and D-state. Indeed, the analysis of several frataxin X-ray structures, from different sources (pdb id: 2fql [17], 1ekg [18], 1ew4 [19]), revealed no interaction between the N-state of the protein and sulfate, chloride or sodium ions, even though these ions are very abundant in the crystallization conditions. Since the protein-solvent interactions involve always water molecules, the same assumption made in the case of pure water should hold in aqueous solutions of NaCl and Na_2_SO_4_. It is well known that also the Na^+^, Cl- and SO42- ions can be bound by some globular proteins due to specific structural and electrostatic features of the binding sites [20]. The present approach, however, cannot account for such binding effects on the conformational stability of globular proteins.

As a consequence of the above assumptions, the ΔGd expression, in both water and aqueous salt solutions, becomes:

$$\Delta G_{d}=\Delta \Delta G_{c}-T\cdot \Delta S_{conf}$$ (2)

Eq (2) looks like the protein stability scenario proposed by Kauzmann [21]. ΔΔGc is an entropic quantity [22] and it represents the loss in translational freedom of solvent molecules due to the solvent-excluded volume increase upon denaturation. Thus, it is always a stabilizing factor for the N-state [9, 10]. The increase in solvent-excluded volume is strictly correlated to the increase in water accessible surface area, WASA [23], upon denaturation [24]. Numerical estimates for the quantities appearing in Eq (2) have to be provided to shed light on the conformational stability increase caused by the addition of NaCl or Na_2_SO_4_ to water.

## Calculation Procedure

A sphere of radius *a* = 15 Å is selected to model the N-state, whereas three prolate spherocylinders, with different values of radius (*a*) and cylindrical length (*l*), are selected to model the D-state (this should be important to test the “robustness” of the model). The spherocylinder sizes are: (1) *a* = 6.0 Å and *l* = 117.0 Å for D-state I; (2) *a* = 5.34 Å and *l* = 150.7 Å for D-state II; (3) *a* = 5.0 Å and *l* = 173.3 Å for D-state III. All these objects (representing the N-state and D-states) have the same van der Waals volume (VvdW = 14137 Å3), but a markedly different water accessible surface area (WASA). A summary of the geometric properties of the sphere and the spherocylinders is reported in Table 1 (see also S2 Text). These numbers correspond to a 138-residue globular protein, since the van der Waals volume of an average residue is 102.5 Å3 [9], and should be reliable for a comparison with Yfh1, that consists of 123 residues. It is worth noting that detailed Monte Carlo simulations by Tran and Pappu (accounting exclusively for the repulsive interactions among residues) indicate that average shapes of the D-state for 23 globular proteins are consistent with prolate ellipsoids [25]. The latter are similar to the prolate spherocylinders considered in the present work [9, 10, 16].

**Table 1:** Main geometric properties of the sphere representing the N-state and the three spherocylinders approximating the D-state.

	radius, *a* (Å)	cylindrical length, *l* (Å)	V_vdw_ (Å^3^)	WASA (Å^2^)
N-state	15	- -	14137	3380
D-state I	6.00	117.0	14137	6128
D-state II	5.34	150.7	14137	6952
D-state III	5.00	173.3	14137	7485

**Fig 1:**
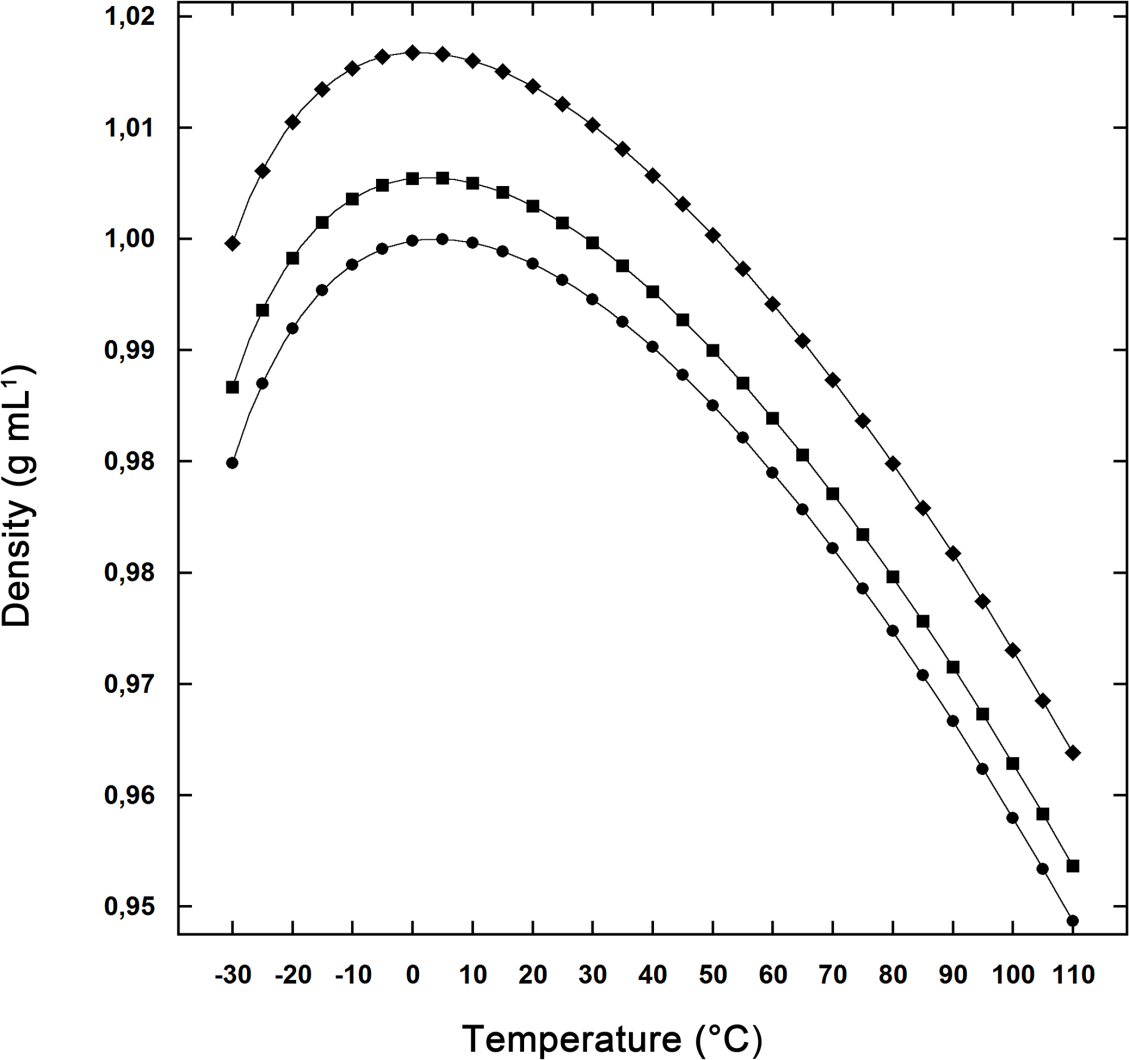
Pure water and solution densities. Density of pure water (circles), 0.1 m NaCl solution (squares), and 0.1 m Na_2_SO_4_ (rhombi) as a function of temperature.

[9]Once the dimensions of the sphere and of the spherocylinder have been fixed, the ΔΔGc quantity is calculated using classic scaled particle theory (SPT) [26, 27]. The ΔΔGc quantity proves to be always a large and positive number, stabilizing the N-state [9, 10, 16, 24], because the two cavities, even possessing the same VvdW, cause a markedly different solvent-excluded volume effect. This effect is markedly larger for the D-state simply because WASA(D-state) > WASA(N-state). Calculations have been carried out at P = 1 atm, over a large temperature range (from -30°C to 70°C), for water, 0.05 m and 0.1 m NaCl, 0.05 m and 0.1 m Na_2_SO_4_ aqueous solutions. Experimental values of the density have been used [28, 29]. Actually, the numerical equations provided by Millero and co-workers for the two salt solutions, representing experimental data above 0°C, have been considered correct down to -30°C [28, 29]. A comparison between the density of water and that of the two 0.1 m salt solutions is reported in Fig 1 (note that molality is preferred to molarity because the solution density depends upon temperature). Since the density of aqueous salt solutions plays a fundamental role in the present approach, it is necessary to take into account the uncertainty associated with experimental density values. The latter uncertainty amounts to 0.05% of the reported average values [29]. Classic SPT calculations have also been performed at the two density extremes for each temperature to test the “robustness” of the results.

Moreover, the following hard sphere diameters, assumed to be temperature-independent, have been used: σ = 2.80 Å for H2O molecules; 2.02 Å for Na+ ions; 3.62 Å for Cl- ions; 4.60 Å for SO42- ions [15, 30–33].

Assuming that the D-state conformational features are not affected by the presence of salts, the magnitude of the T ΔSconf contribution should not to change in passing from water to aqueous salt solutions. If each residue of the protein chain gains an average, temperature-independent conformational entropy upon denaturation [9, 10, 16], it is possible to write:

$$T\cdot \Delta S_{conf}=T\cdot N_{res}\times \Delta S_{conf}\left(res\right)$$ (3)

where Nres = 138, and the T ΔSconf contribution proves to be a straight line. The following values have been selected for ΔSconf(res): 19.1 J K-1 mol-res-1 for D-state I, 24.4 J K-1 mol-res-1 for D-state II and 27.8 J K-1 mol-res-1 for D-state III. The ΔSconf(res) term is considered to increase on lengthening the spherocylinder that models the D-state since the conformational freedom of the chain should increase (i.e., keeping VvdW fixed, the length of the spherocylinder is a measure of the D-state compactness, and the latter should be a measure of the conformational freedom of the chain). In this respect, it is worth noting that Sosnick and co-workers [34] have recently been able to obtain a direct estimate of ΔSconf(res) for ubiquitin producing reliable statistical ensembles for both the N-state and D-state, by means of very long MD trajectories. The average value obtained by Sosnick and co-workers is 19.5 J K-1 mol-res-1 at 300 K. The numbers used in the present analysis are in line with the latter value and other literature estimates [35–37].

## Results

**Fig 2:**
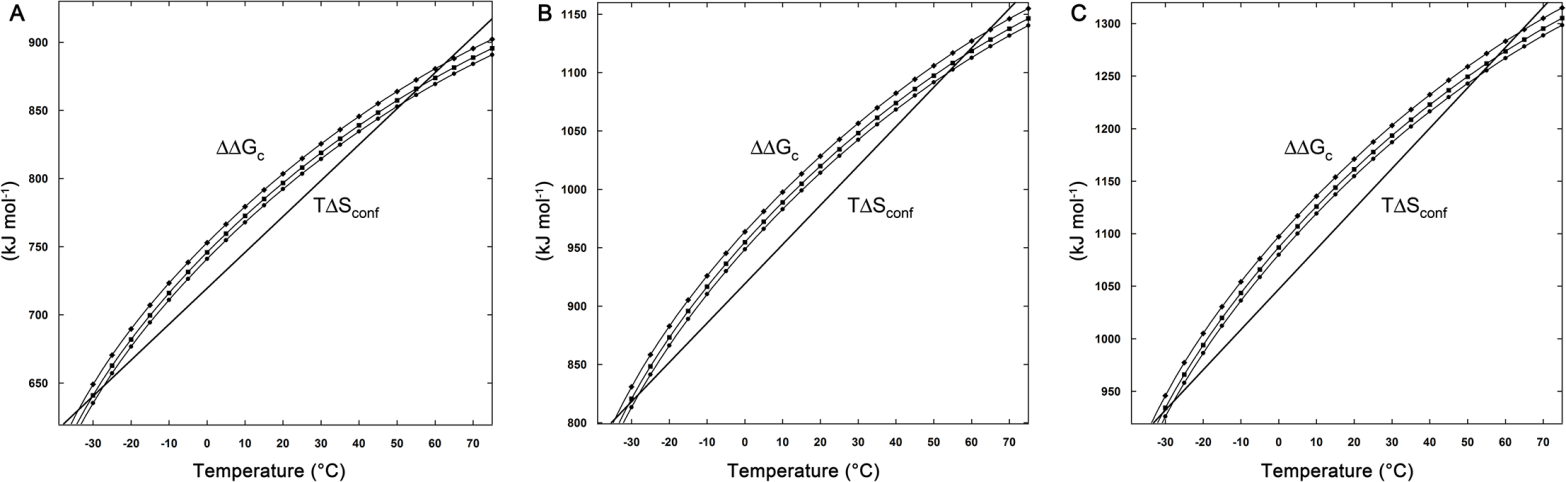
∆∆Gc curves for the three D-state cases. The curves ∆∆Gc = ∆Gc(D-state)—∆Gc(N-state) for pure water (circles), 0.1 m NaCl (squares), and 0.1 m Na_2_SO_4_ (rhombi) as a function of temperature are shown together with the T ΔSconf straight line calculated fixing Nres = 138. (A) D-state I with ΔSconf(res) = 19.1 J K-1 mol-res-1. (B) D-state II with ΔSconf(res) = 24.4 J K-1 mol-res-1. (C) D-state III with ΔSconf(res) = 27.8 J K-1 mol-res-1. The dimensions of the three D-states are reported in Table 1.

The profile of the functions ΔΔGc(H2O), ΔΔGc(0.1 m NaCl), ΔΔGc(0.1 m Na_2_SO_4_) and T ΔSconf, calculated in the temperature range from -30 to 70°C, is shown in Fig 2 for all the considered cases. A qualitatively similar trend is obtained in the 0.05 m salt solutions; data not shown. The larger is ∆WASA (defined as WASA(D-state)—WASA(N-state)) the larger is the value of ∆∆Gc; ∆WASA is in fact a measure of the rise in solvent-excluded volume effect associated with chain unfolding. More importantly, the Gc functions show a parabola-like profile, which originates from the peculiar temperature dependence of aqueous solution densities (see Fig 1 for the densities of pure water and 0.1 m salt solutions). Indeed, while the density of a common liquid increases on decreasing the temperature, water shows a temperature of maximum density (TMD) at 4.0°C. The TMD value of salt solutions depends upon the salt type and concentration and it is always lower than that of pure water [38, 39]. In particular, TMD is 2.5°C for the 0.1 m NaCl solution, and 1.0°C for the 0.1 m Na_2_SO_4_ solution [39]. The TMD values of all the considered solutions are listed in Table 2. All the ∆∆Gc functions decrease on lowering the temperature as a direct consequence of both the density decrease and the decrease in random thermal energy of the solvent particles bombarding the cavity surface (i.e., the RT factor present in all the formulas to calculate the work of cavity creation [26, 27]).

**Table 2:** Some features of all the considered solutions at 25°C and 1 atm, and their TMD values are reported. Cold and hot denaturation temperatures, together with the ΔGd at the temperature of maximal stability, for the D-state II case, are listed in the last three lines.

	Water	NaCl 0.05 m	NaCl 0.1 m	Na_2_SO_4_ 0.05 m	Na_2_SO_4_ 0.1 m
density at 25°C [g mL-1]	0.9970	0.9991	1.0011	1.0034	1.0097
ξ3 at 25°C	0.3831	0.3836	0.3842	0.3846	0.3861
total number density at 25°C [mol L-1]	55.34	55.40	55.43	55.45	55.55
TMD [°C]	4.0	3.5	2.5	2.5	1.0
Td,cold [°C]	-28	-29.5	-31	-31.5	-34.5
Td,hot [°C]	53.5	56	58	59	64
∆Gd(Tmax) [kJ mol-1]	30	33	36	38	45

**Fig 3:**
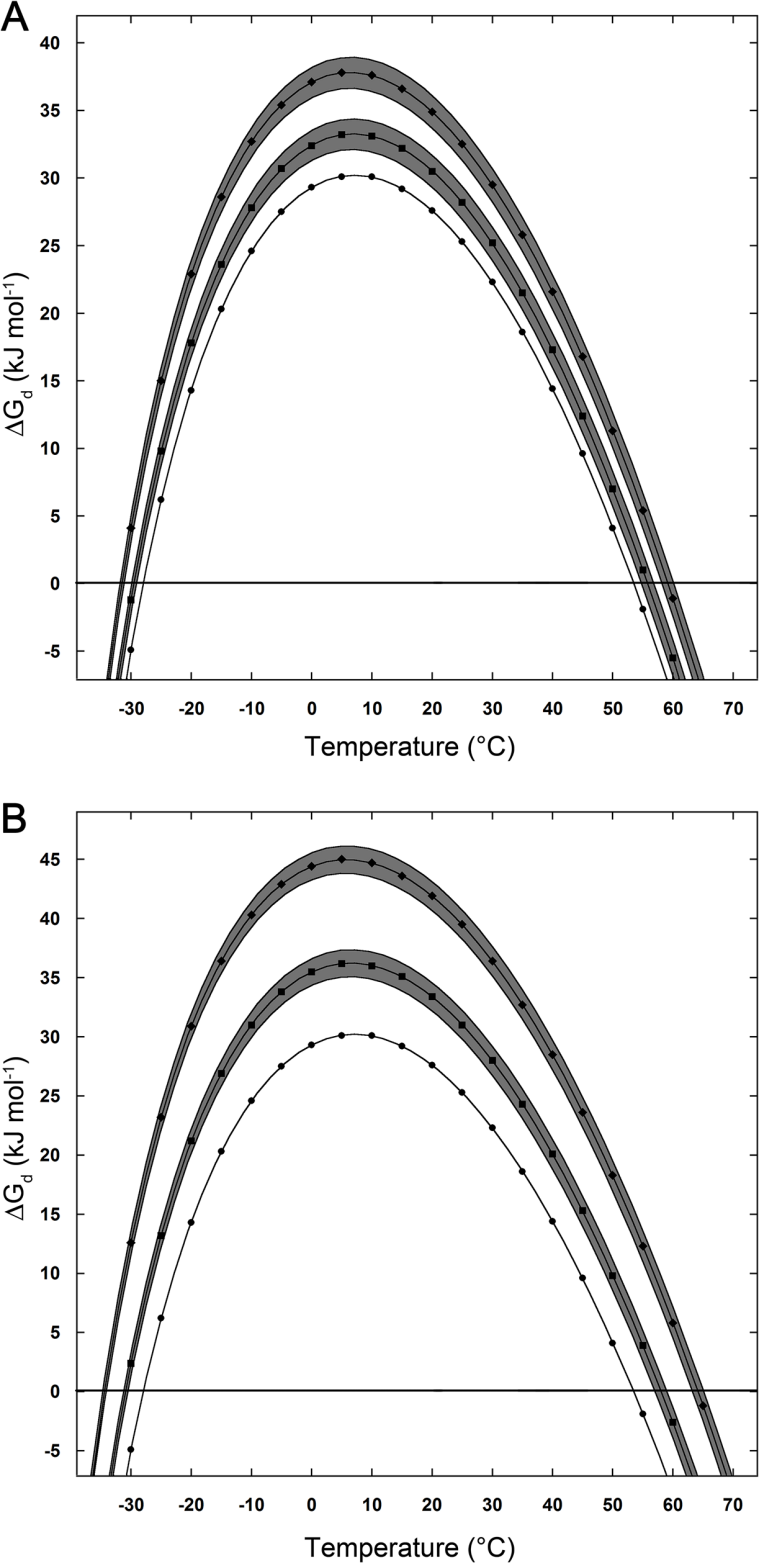
Stability curves for D-state II case. Thermodynamic stability curves of the model globular protein, considering the D-state II case, and taking into account the uncertainty in the density of the salt solutions; see text for further details. (A) pure water (circles), 0.05 m NaCl (squares) and the shaded area, 0.05 m Na_2_SO_4_ (rhombi) and the shaded area. (B) pure water (circles), 0.1 m NaCl (squares) and the shaded area, 0.1 m Na_2_SO_4_ (rhombi) and the shaded area.

The two points of intersection between the–T ΔSconf straight line and the ΔΔGc curves correspond to Td,cold (on the "cold side") and to Td,hot (on the "hot side"), respectively. Cold and hot denaturation temperatures for all the investigated solutions, together with the ΔGd(Tmax) values, are reported in Table 2 for the D-state II case. The calculated thermodynamic stability curves, ΔGd versus temperature, are shown in Fig 3A for the 0.05 m salt solutions, and in Fig 3B for the 0.1 m salt solutions, in comparison to that holding in pure water, referring to the D-state II case. These curves also show the effect due to the uncertainty associated with the density of salt solutions (no error is associated with water density in view of the precision of the data reported by Kell [28]). The stability curves referring to the case of D-state I and D-state III are qualitatively similar and are reported as Supporting Information. The main features of these curves prove to be “robust” to density uncertainty, to the different D-state spherocylinders and to ΔSconf(res) numbers. The shift of the cold and hot denaturation temperatures and of the ΔGd(Tmax) value increases on passing from 0.05 m to 0.1 m aqueous salt solutions. The values reported in Table 2, always referring to the case of D-state II, indicate that, even small concentrations of the two salts, lead to a significant stability increase of the model protein. Specifically: (a) Td,cold = -28°C in water, -31°C in 0.1 m NaCl, -34.5°C in 0.1 m Na_2_SO_4_; (b) Td,hot = 53.5°C in water, 58°C in 0.1 m NaCl, 64°C in 0.1 m Na_2_SO_4_; (c) ΔGd(Tmax) = 30 kJ mol-1 in water, 36 kJ mol-1 in 0.1 m NaCl, and 45 kJ mol-1 in 0.1 m Na_2_SO_4_. The obtained value of ΔGd(Tmax) in water is absolutely consistent with a stable globular protein, corresponding to a stabilization Gibbs energy of about 220 J mol-res-1, in line with experimental data [40]. Also the obtained Tmax value, about 8°C in all the investigated cases (see Fig 3), is in agreement with the average value determined over a large set of globular proteins, Tmax = 283 ± 20 K [40]. The present results are in line with experimental findings on Yfh1 [8].

**Fig 4:**
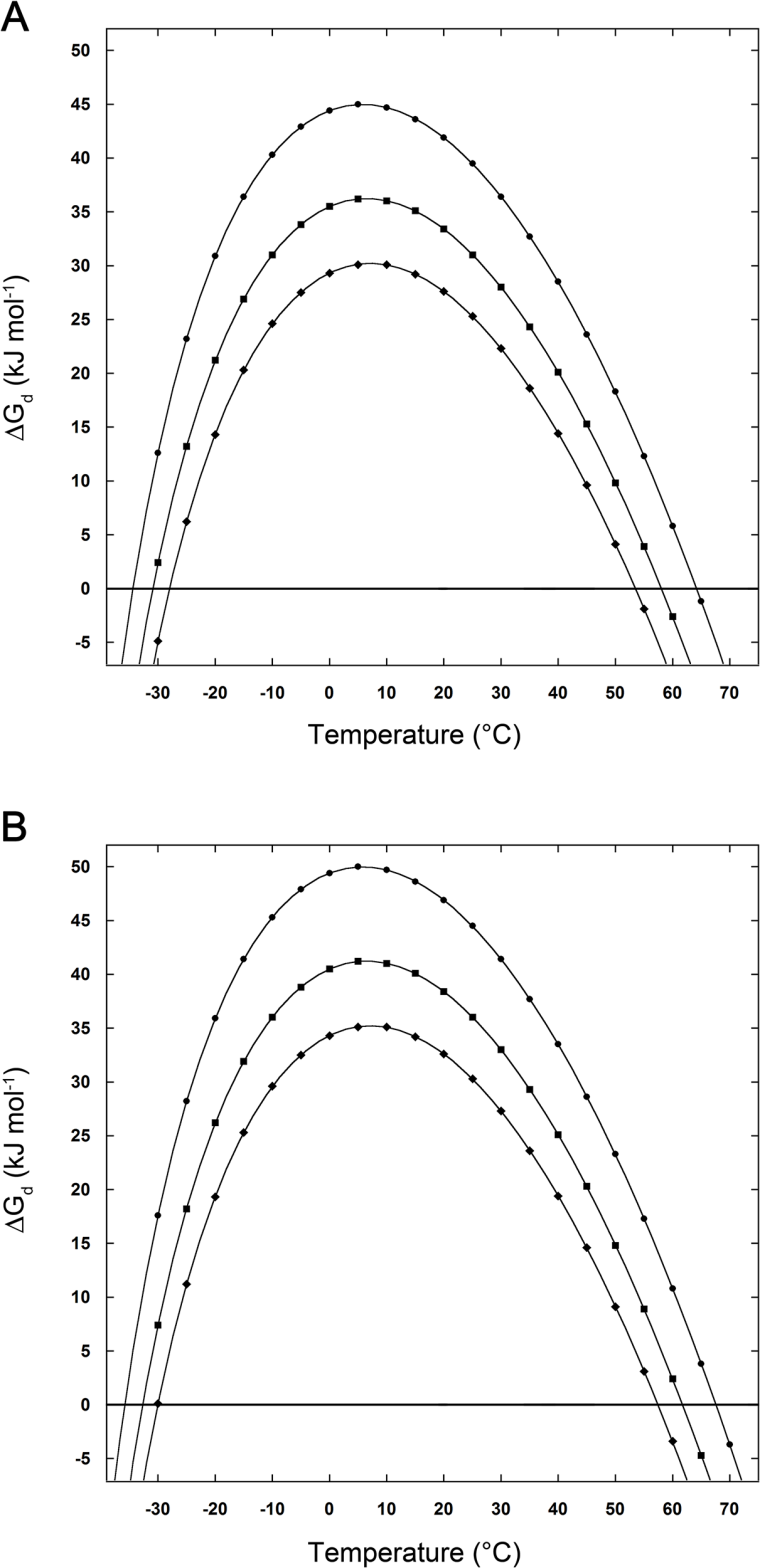
Effect of ∆E on stability curves. Thermodynamic stability curves of the model globular protein, considering the D-state II case, with (A) ∆E = 0 and (B) ∆E = 5 kJ mol-1. See text for further details.

As a final check, it is important to assess the effect of removing the assumption ΔE = 0 on the obtained results. The ΔE term should be a positive quantity in order to stabilize the N-state and should not be affected by the presence of NaCl or Na_2_SO_4_ because the corresponding ions interact preferentially with water molecules, not with protein surface groups. By fixing ΔE = 5 kJ mol-1, temperature-independent and salt-independent, the obtained stability curves are shown in Fig 4 for the case of D-state II and 0.1 m salt solutions. It is evident that the qualitative trend does not change because the positive and constant ∆E quantity causes a lift-up of all the parabola-like curves. It is worth noting that the ∆E quantity should depend slightly on temperature because the strength of both protein-water and intra-protein interactions changes little with temperature. This strength, in fact, depends upon the distance between the interacting groups, and this distance should change slightly in view of the very small temperature dependence of the density of both water and aqueous salt solutions [28, 29].

## Discussion

To the best of our knowledge, the salt effect on cold denaturation has been investigated only in the case of yeast frataxin, Yfh1 [8]. Therefore, the experimental results on Yfh1 motivated the present analysis. The latter, however, having a statistical mechanical ground, is not aimed to quantitatively reproduce the results obtained in the case of Yfh1, but to provide a general and qualitative rationalization of the stabilization afforded by small concentrations of NaCl or Na_2_SO_4_. In this respect, it is worth noting that these two salts have shown a similar shift of the collapse transition temperature (akin to the cold denaturation temperature) in the case of the uncharged poly(*N*-isopropylacrylamide), PNIPAM, and elastin-like polypeptides [41].

The stability increase of the model protein is due to the ∆∆Gc magnitude that is larger in the aqueous salt solutions with respect to the pure water case (see Fig 2). The liquid density and the liquid particle size are the fundamental quantities to determine the ΔGc magnitude, according to both classic SPT and computer simulations [9, 10, 15, 16, 42–48]. In the present case, the size does not play a role because the average diameter of liquid particles is 2.8 Å in both water and all the considered aqueous salt solutions. In contrast, the experimental densities of 0.05 m and 0.1 m aqueous solutions of both NaCl and Na_2_SO_4_ are larger than that of water over the whole considered temperature range (see Fig 1, for instance). As a consequence, the values of the volume packing density, ξ 3, which is the fraction of the liquid volume really occupied by solvent molecules and ions, of both aqueous salt solutions prove to be larger than that of water at any temperature (i.e., ξ3 = 0.3831 in water, 0.3842 in 0.1 m NaCl, and 0.3861 in 0.1 m Na_2_SO_4_, at 25°C). An increase of ξ 3 leads to (a) a decrease in the fraction of void volume in the liquid, (b) a decrease in the probability of finding a molecular-sized cavity in the liquid volume, and (c) an increase in the ∆Gc magnitude [15, 16]. In addition, the higher density of aqueous salt solutions translates in a higher total number density (i.e., number of moles per liter), leading to an increase in the magnitude of the solvent-excluded volume effect. This is why, even though the ξ 3 values are very close, such as in the case of water and 0.05 m NaCl, the ∆∆Gc contribution is larger in the salt solution. Although the difference in total number density and in ξ3 values is small, the impact on the ∆∆Gc magnitude is significant because the effect is amplified by the large difference in WASA of the considered cavities. The electrostatic charge-dipole interactions, strengthened by the high charge density of the considered ions, are responsible, at a molecular level, of the density increase caused by the addition of NaCl or Na_2_SO_4_ to water.

The assumption that the structural-geometric features of both the N-state and D-state are not affected by the presence of small concentrations of NaCl or Na_2_SO_4_ implies that the ΔSconf(res) magnitude should not depend upon the presence of such salts. Of course, the outcomes of the approach are very sensitive to the value assigned to ΔSconf(res), but this should not detract from the general qualitative nature of the emerged stabilization mechanism.

The simplified nature of the present geometric models of both the N-state and D-state should not be forgotten. No charge is considered to exist on the surface of the models (in contrast, an important and peculiar feature of Yfh1 [20]) and this implies that the present approach cannot take into account: (a) the effect of attractive-repulsive charge-charge interactions; (b) the screening effect of electrostatic interactions provided by a high ionic strength in aqueous salt solutions. However, the approach has been used to devise a rationalization of the effect that small concentrations of NaCl or Na_2_SO_4_ have on the stability of the model protein.

The NaCl and Na_2_SO_4_ effect on the conformational stability of the model protein follows the Hofmeister series [49], which sorts ions on the basis of their ability to increase the stability of globular proteins [50]. In particular, Na+, Cl- and SO42- are classified as stabilizing ions, the latter being one of the most stabilizing anions of the series. The molecular-level origin of the Hofmeister series is still not clear and contrasting ideas have been proposed [51–54]. The present theoretical approach indicates that: (a) the stability increase of the model protein is a direct consequence of the higher density of salt solutions, which originates from the strong electrostatic interactions between ions and water molecules; (b) there is no need to consider the effects of ions on the structural features of water (i.e., no need to classify ions in structure-breaking, chaotropic, or structure-making, kosmotropic, ones) [53].

A final point. Several authors [55, 56] have claimed that ∆Gc is directly proportional to the liquid-vapor surface tension, γ∞, of the solvent. The addition of either NaCl or Na_2_SO_4_ to water causes an increase of γ∞ [50], and so also this explanation seems to be right. However, the experimental values of γ∞ of water show a continuous decrease over the temperature range from -25 to 100°C [57]. This continuous decrease markedly contrasts with the parabola-like temperature dependence of water density that translates in the parabola-like temperature dependence of ∆Gc in water. The latter is a feature of water emerged both in classic SPT calculations [9, 10, 24], and molecular dynamics simulations in reliable water models [58, 59].

In conclusion, the present approach is able to explain the ability of NaCl and Na_2_SO_4_ to stabilize globular proteins, causing an overall increase of the thermodynamic stability curve, leading to a lower Td,cold and a higher Td,hot. The theoretical model works well without the need to introduce *ad hoc* assumptions for the ion effects, confirming its reliability. A fundamental role is played by the solution density increase upon addition of salts to water, which leads to an increase in the stabilizing ∆∆Gc contribution. The higher the solution density, the more amplified the solvent-excluded volume effect will be.

## Supporting Information

S1 Fig Stability curves for D-state I case. Thermodynamic stability curves of the model globular protein, considering the D-state I case, and taking into account the uncertainty in the density of the salt solutions; see text for further details. (A) pure water (circles), 0.05 m NaCl (squares) and the shaded area, 0.05 m Na_2_SO_4_ (rhombi) and the shaded area. (B) pure water (circles), 0.1 m NaCl (squares) and the shaded area, 0.1 m Na_2_SO_4_ (rhombi) and the shaded area.
(TIF)

S2 Fig Stability curves for D-state III case. Thermodynamic stability curves of the model globular protein, considering the D-state III case, and taking into account the uncertainty in the density of the salt solutions; see text for further details. (A) pure water (circles), 0.05 m NaCl (squares) and the shaded area, 0.05 m Na_2_SO_4_ (rhombi) and the shaded area. (B) pure water (circles), 0.1 m NaCl (squares) and the shaded area, 0.1 m Na_2_SO_4_ (rhombi) and the shaded area.
(TIF)

S1 Text On the ∆E = 0 assumption. (DOCX)

S2 Text Volume change upon protein denaturation. (DOCX)
