# Supplementary figures and images for: On the Effect of Sodium Chloride and Sodium Sulfate on Cold Denaturation
Source: PLoS One. 2015 Jul 21;10(7):e0133550. doi: 10.1371/journal.pone.0133550 (PMC4511003; doi:10.1371/journal.pone.0133550)

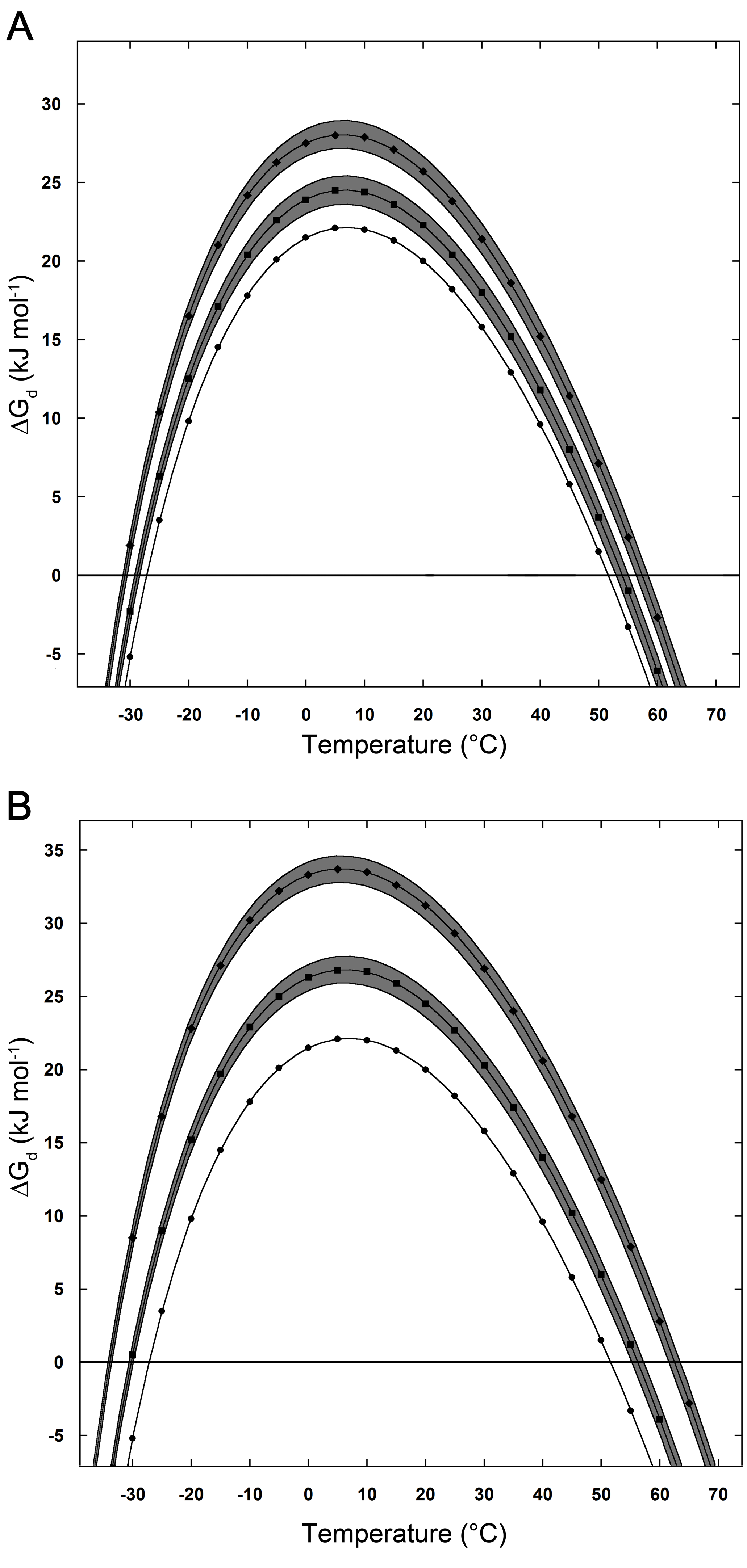

Supplement: S1 Fig — Thermodynamic stability curves of the model globular protein, considering the D-state I case, and taking into account the uncertainty in the density of the salt solutions; see text for further details. (A) pure water (circles), 0.05 m NaCl (squares) and the shaded area, 0.05 m Na2SO4 (rhombi) and the shaded area. (B) pure water (circles), 0.1 m NaCl (squares) and the shaded area, 0.1 m Na2SO4 (rhombi) and the shaded area. (TIF) [file pone.0133550.s001.tif]

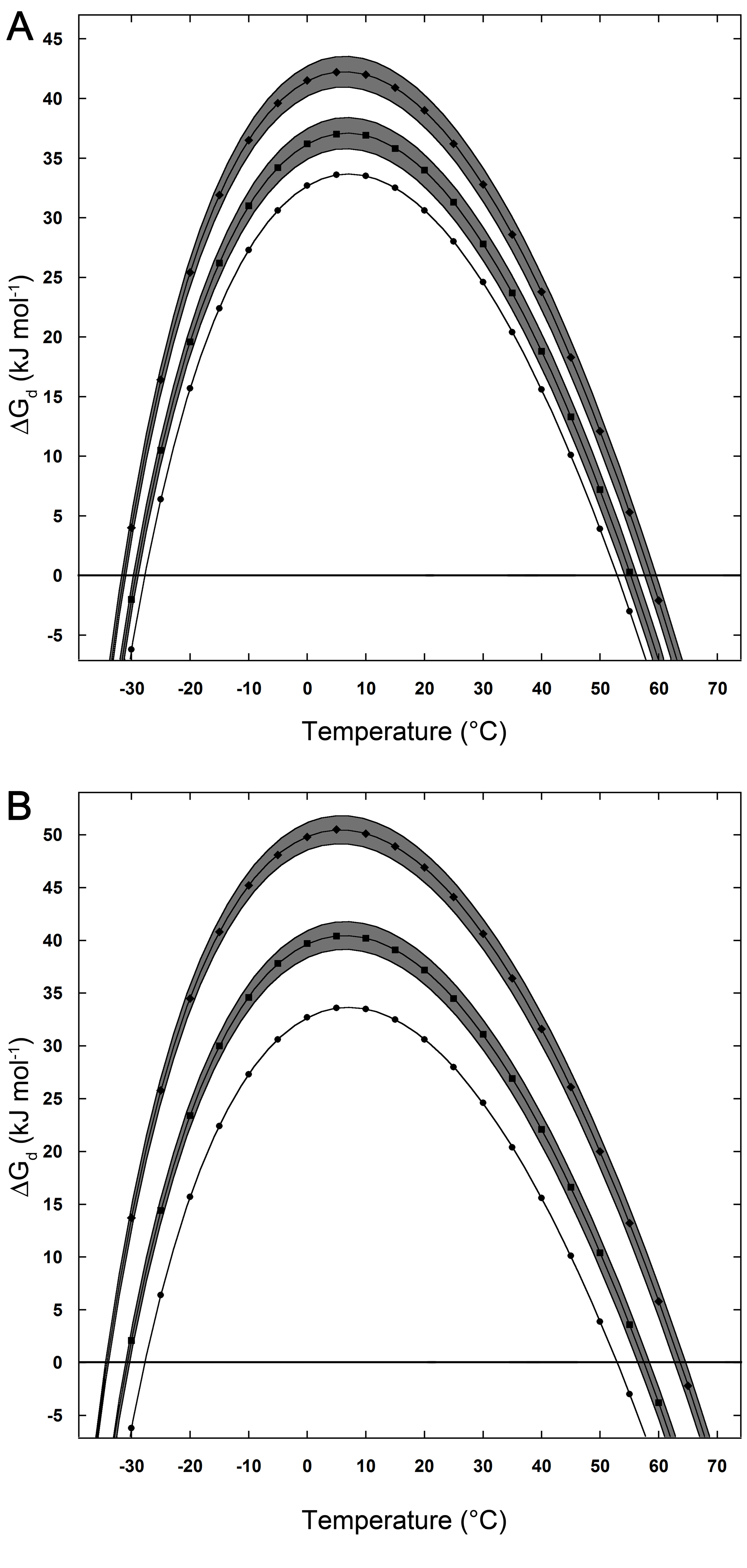

Supplement: S2 Fig — Thermodynamic stability curves of the model globular protein, considering the D-state III case, and taking into account the uncertainty in the density of the salt solutions; see text for further details. (A) pure water (circles), 0.05 m NaCl (squares) and the shaded area, 0.05 m Na2SO4 (rhombi) and the shaded area. (B) pure water (circles), 0.1 m NaCl (squares) and the shaded area, 0.1 m Na2SO4 (rhombi) and the shaded area. (TIF) [file pone.0133550.s002.tif]
