# Supplementary material for: On the Effect of Sodium Chloride and Sodium Sulfate on Cold Denaturation
Source: PLoS One. 2015 Jul 21;10(7):e0133550. doi: 10.1371/journal.pone.0133550 (PMC4511003; doi:10.1371/journal.pone.0133550)
Supplement: S1 Text — (DOCX) [file pone.0133550.s003.docx]

# Supporting Information

**S1 Text. On the** Δ**E = 0 assumption**

In a recent and interesting study, Das and Matysiak [1] have shown that a hydrophobic 32-bead polymer is in a collapsed state at 400 K and 1 atm, in a coarse-grained model of water, and in a swollen state at 250 K and 1 atm, in the same water model (i.e., the model hydrophobic polymer shows the phenomenon of cold denaturation). In the MD simulations the energy of a single monomer-monomer contact is fixed equal to the energy of a single monomer-water contact. The number of monomer-water contacts proves to be about 130 in the swollen state, and about 90 in the collapsed state. The number of monomer-monomer contacts in the collapsed state, more-or-less, closes the gap between the above two values. The Das and Matysiak results should be considered an indication that the ΔE = 0 assumption is not unreliable, by recognizing that the N-state of globular protein is marginally stable.

**References**

1. Das P, Matysiak S (2012) Direct characterization of hydrophobic hydration during cold and pressure denaturation. J Phys Chem B 116: 5342-5348.
