# Supplementary material for: On the Effect of Sodium Chloride and Sodium Sulfate on Cold Denaturation
Source: PLoS One. 2015 Jul 21;10(7):e0133550. doi: 10.1371/journal.pone.0133550 (PMC4511003; doi:10.1371/journal.pone.0133550)
Supplement: S2 Text — (DOCX) [file pone.0133550.s004.docx]

# Supporting Information

**S2 Text. Volume change upon protein denaturation**

It is firmly established that the volume change associated with the denaturation of small globular proteins, ΔVd, is an astonishingly small and negative quantity [1,2]. This is why the N-state and the D-state of the model globular protein are constructed to possess the same VvdW. This is important also to appreciate that it is not possible to directly use the values of the radius of gyration, RG, experimentally determined for some globular proteins, in both the N-state and the D-state. In fact, using such RG values, the D-state should be modelled by a sphere with a radius significantly larger than that of the sphere modelling the N-state. This procedure would lead to a scenario not compatible with the experimental finding that ΔVd is an astonishingly small and negative quantity.

**References**

1. Royer CA (2002) Revisiting volume changes in pressure-induced protein unfolding. BBA-Protein Struct M 1595: 201-209.

2. Chalikian TV (2003) Volumetric properties of proteins. Annu Rev Biophys Biomol Struct 32: 207-235.
